# A decade of systematic reviews: an assessment of Weill Cornell Medicine's systematic review service

**DOI:** 10.5195/jmla.2023.1628

**Published:** 2023-07-10

**Authors:** Michelle R. Demetres, Drew N. Wright, Andy Hickner, Caroline Jedlicka, Diana Delgado

**Affiliations:** 1 mrd2006@med.cornell.edu, Samuel J. Wood Library & C.V. Starr Biomedical Information Center, Weill Cornell Medicine, New York, NY; 2 drw2004@med.cornell.edu, Samuel J. Wood Library & C.V. Starr Biomedical Information Center, Weill Cornell Medicine, New York, NY; 3 alh4014@med.cornell.edu, Samuel J. Wood Library & C.V. Starr Biomedical Information Center, Weill Cornell Medicine, New York, NY; 4 cmj4001@med.cornell.edu, Samuel J. Wood Library & C.V. Starr Biomedical Information Center, Weill Cornell Medicine, New York, NY; 5 did2005@med.cornell.edu, Samuel J. Wood Library & C.V. Starr Biomedical Information Center, Weill Cornell Medicine, New York, NY

**Keywords:** Evidence synthesis, systematic reviews, meta-analysis, library services, research services

## Abstract

**Background:**

The Weill Cornell Medicine, Samuel J. Wood Library's Systematic Review (SR) service began in 2011, with 2021 marking a decade of service. This paper will describe how the service policies have grown and will break down our service quantitatively over the past 11 years to examine SR timelines and trends.

**Case Presentation:**

We evaluated 11 years (2011-2021) of SR request data from our in-house documentation. In the years assessed, there have been 319 SR requests from 20 clinical departments, leading to 101 publications with at least one librarian collaborator listed as co-author. The average review took 642 days to publication, with the longest at 1408 days, and the shortest at 94 days. On average, librarians spent 14.7 hours in total on each review. SR projects were most likely to be abandoned at the title/abstract screening phase. Several policies have been put into place over the years in order to accommodate workflows and demand for our service.

**Discussion:**

The SR service has seen several changes since its inception in 2011. Based on the findings and emerging trends discussed here, our service will inevitably evolve further to adapt to these changes, such as machine learning-assisted technology.

## BACKGROUND

Best practice standards and previous studies have emphasized the importance of the librarian in the systematic review (SR) process [1–4]. Librarians involved in SRs take on a plethora of roles outside the traditional expert searcher, including but not limited to citation management, collaboration and planning, question formulation, reporting and documentation, protocol development, and assistance with technological and analytical tools [5]. As identified by Townsend et al., librarians must also master and leverage multiple competencies and their associated skills and knowledge pieces in order to provide these services [6]. This complex involvement in SRs and other evidence synthesis projects often necessitates the development of a formal service, which many institutions and libraries have implemented and documented [7,8].

SR services can face several issues in implementation, collaboration challenges, and scalability with offerings, as well as many other institutional-specific concerns [9–11]. One particularly important issue is librarian capacity. As McKeown & Ross-White address in their 2019 study, defining collaboration from the outset is essential [9].

While service models can be standardized, creating different levels of service for different patrons may be necessary to maximize librarians' return on investment.

Librarian time spent on SRs has been documented and can vary widely depending on the task and individual librarian [12]. Bullers et al. found average aggregated time spent on standard tasks was 26.9 hours, with a median of 18.5 hours [12]. With many of the librarian's roles, and therefore much of their effort, being concentrated at the start of the project [13], the decision to abandon an SR often happens after librarian time has already been contributed. Therefore, developing and evolving a SR service into one that supports both patron and provider can be challenging. Tracking and documenting an SR service's outputs is crucial to this effort; however, traditional or existing library metrics may be insufficient for this task [9].

## CASE PRESENTATION

The Weill Cornell Medicine (WCM) Samuel J. Wood Library's SR service began in 2011, with 2021 marking a decade of service. The service started with two requests in its first year and expanded to a team of eight SR librarians tackling 60 requests in 2021. The service now supports a variety of evidence synthesis types including scoping and rapid reviews, guidelines, and consensus statements. We will describe how the service policies have grown and will break down our service quantitatively over the past 11 years to examine SR timelines and trends.

We evaluated 11 years (2011-2021) of SR request data from our in-house documentation. Data included information available from the SR request form as well as each SR librarian's self-reported progress, all continuously documented in a shared Excel spreadsheet. Both the request form and the way progression data were recorded changed several times over the course of the 11 years. As a result, some SR requests recorded prior to 2015 contain incomplete data.

We examined data from all teams within the timeframe requesting a formal collaboration. This excludes our advisory service for students conducting SRs as part of their educational programs. All SR collaborations are free-of-charge, apart from potential InterLibrary Loan (ILL) fees discussed later in this paper. A formal SR collaboration can include the following:

- Helping define a research question
- Assisting in developing and registering the protocol
- Selecting specific databases and other resources to be searched
- Developing database-specific search strategies using a combination of keywords and controlled vocabulary to maximize precision and recall
- Conducting literature searches
- Snowballing - pulling references from bibliographies, pulling “cited by” references and identifying related articles
- Delivering results into a bibliographic management tool such as EndNote or Mendeley
- Performing search updates in selected databases
- Writing methods section of manuscript
- Suggesting journals relevant to areas of research
- Recommending Medical Subject Headings (MeSH) and Emtree terms and keywords for articles
- Providing access to Covidence, a systematic review tool [14]

### Publications

As a requirement of using WCM's SR service as a formal collaboration, co-authorship is required for all librarian collaborators on any resulting manuscripts. In the years assessed, there have been 319 SR requests from 20 clinical departments, leading to 101 publications with at least one librarian collaborator listed as coauthor. Among the SR teams that published papers, the majority of teams used more than half of our offered services as part of their formal collaboration.

### Timelines

The SR process is time consuming, with Cochrane Collaboration's timeline for a review suggesting 12 months or more [3]. Based on our documentation data, the average review took 642 days to publication, with the longest being 1408 days and the shortest being 94 days.

**Table 1:** Timelines for WCM's SR service

	Time to Methods Written (Days)	Time to Paper Submitted (Days)	Time to Paper Published (Days)
Shortest	18	42	94
Average	216	295	642
Standard Deviation	179	195	602
Longest	961	930	1408

To support these requests, time spent by the librarian varied. On average, librarians spent 14.7 hours in total on each review. Published reviews saw librarians spending an average of 16.9 hours on each review. Unpublished reviews averaged 12.2 hours of librarian time. In comparison, Bullers et al. found a median of 18.4 hours spent on “standard tasks” [12]. It should be noted that more time spent does not necessarily relate to completion, as unpublished reviews may have less time spent due to unfinished steps in the review, such as snowballing and manuscript writing. We did not find any meaningful differences in review type (SR vs other evidence synthesis types), discipline (clinical department), requesting team size, or previous experience with the SR service.

### SR teams

Our data showed that of the 101 published papers, repeat SR requesters (i.e., the requester has worked on another SR formal collaboration with the service) published a total of 43 papers.

There were 42 repeat SR requesters who submitted 117 requests.

Our SR request form requires the submitter to list team members. A fundamental aspect of SRs is that they cannot be performed alone; multiple members are required in order to limit bias [3]. However, at the request stage, often teams have not yet been cemented. Our data showed that teams with 2 or 3 members at the start were most represented in the number of requests and the number of papers published.

**Table 2:** Size of requesting teams and final completed SR project stage

Size of Team	Total Requests	Methods Written	Paper Submitted *	Paper Published**
1	75	7	6	14
2	79	17	9	19
3	89	24	15	20
4	52	14	5	9
5	15	2	1	1
6	7	1	1	1
7	1	0	0	1
8	1	0	0	0

* Includes both currently active papers waiting for peer review and papers that did not make it past the submission to the acceptance phase.

** For the 36 papers not included, we did not have full monthly data to report with regard to stages.

### Unfinished projects

Librarians often have little control over the completion of SR projects. However, because most of the librarian's work is at the start of the project, time and effort spent is often the same regardless of whether a SR project gets published or abandoned. Our data showed that SR projects were most likely to be abandoned at the title/abstract screening phase, often after the librarian's largest contribution has already been completed. In attempt to curb the number of abandoned projects, scheduled email check-ins with the SR requesting team at various points in the process have been recommended by our SR librarians. However, we do not have data to support their efficacy.

**Table 3:** Stage of unpublished SR projects

Librarian contribution	Review phase	Number of officially abandoned projects at this stage (% of total projects)
Initial search delivered	Search development	16 (5.0%)
Full search results delivered / uploaded	Title/abstract screening	97 (30.0%)
Full-text delivered / uploaded	Full-text screening	79 (24.7%)
	Data extraction	20 (6.2%)

### Policies

Apart from the story the quantitative data can tell, there are several important policies that have been put into place over the years in order to support workflow and demand.

An important aspect of a SR service is not only defining what the service includes, but also what it does not. In particular, a service must articulate which user groups are outside the service model. For example, while we welcome repeat requesting groups, we typically do not accept two simultaneous review requests from the same group. We ask that the requesting group prioritize one review, and work can begin on the second once the first has entered the data extraction phase. In addition, we instituted a policy in 2019 that our formal collaboration services do not extend to medical or graduate students conducting systematic reviews as part of their educational program. This is similar to the two-tiered model discussed by McKeown & Ross-White [9]. However, librarians can still meet with students to discuss the process and provide guidance throughout, but they will not “do the work.” The SR service for students conducting systematic reviews as part of their educational program can include:

- Referral to relevant guidance documents and reporting standards
- Feedback on framing the research question
- Feedback on initial search strategy
- Advice on relevant electronic databases and sources to search
- Advice on reporting of methods in the manuscript
- Recommendations on where to submit for publication
- Access to Covidence

In response to the demand in these student-led projects during the COVID-19 pandemic (when research with patient groups was halted), we developed a LibGuide outlining the SR process, with links to tools and resources [15]. In addition to these one-on-one guidance instances, systematic review classes are incorporated into educational programs throughout the institution. In 2021, there were 12 systematic review process classes taught in the graduate curriculum and other non-credit/course related guest lectures. This class differentiates between SRs and other review types, articulates their importance in evidence-based practice, and provides an overview of the steps to complete an SR.

Uploading full-text articles to Covidence, which allows SR teams to seamlessly begin the process of full-text screening, has become an important aspect of our service model. However, this has impacted our service in two important ways. Firstly, the volume of full-text to be uploaded by the collaborating librarian is often time prohibitive. To address this, our service has been expanded to include WCM's library assistants; they are added to each Covidence review when necessary and attach full-text or submit ILL requests. This important support team has ranged from 5 to 8 library assistants. Assignments to SRs are dependent on need (i.e., how much full text per review) and the library assistant's availability and current workload. Library assistants were provided with a training class and documentation on an overview of the SR process and working with Covidence software.

The impact of full-text pulling has also impacted our ILL service, which has been previously reported on by other libraries [16]. Occasionally, the demand for ILL requests at this stage can be excessive. Therefore, we have put into place a flat fee ($250 US) that the SR team must pay if there are more than 100 ILL requests. The average cost for 100 articles is around $1,000 US. Because of reciprocity agreements, we pay for approximately 25% of the articles we request, therefore we set the fee at $250 US. This payment from the SR team would only supplement the fees the library has paid; it is not a total reimbursement. This is a relatively new policy, having been implemented in 2021, and we have not yet needed to enforce this.

## DISCUSSION

Our service will inevitably evolve further. For example, if the demand for our service increases past our ability to support it due to fluctuations in SR librarian staffing, we may need to consider policies such as waitlisting. As noted, we did not find any meaningful differences in time-to-completion when considering review type, discipline, requesting team size, or requesting team experience. This makes it impossible to predict how long a project will take to complete and poses challenges for creating policies to address this. Campbell and Dorgan have previously discussed the difficulty of supporting SRs with a limited librarian capacity [11]. Their 8-part strategy lays out a thoughtful plan, some portions of which our service already has in place, such as redefining service policies for external users. However, there are many changes that our service may need to consider, including better organizing search support resources, negotiating with faculty to make systematic review search assignments reasonable, and requiring clients to do advanced preparation for searches, such as protocol completion prior to formal SR collaborations [11]. Sustainability of our service is largely dependent on user demand and SR librarian staffing, things we cannot anticipate. However, implementing workflow changes such as these or the clearly defined team-based service model as outlined by Roth could be useful [17].

Technical support for SRs has changed drastically in the past decade, with the introduction of screening tools such as Covidence and DistillerSR [14,18]. Since our institutional subscription to Covidence began in 2017, our service model has adapted to include support and troubleshooting for this software. Automation tools are continuing to develop, not only in the screening phase of the SR process but also in risk of bias and data extraction phases. Tools such as RobotReviewer aim to “(semi-) automate evidence synthesis using machine learning and natural language processing” [19]. As the scholarly conversation surrounding machine learning assistance grows, it is important to keep current with these developments [20,21]. Indeed, Covidence has implemented machine learning updates in 2022 [22]. The Cochrane Randomized Controlled Trial (RCT) classifier has been integrated into the software, using machine learning to tag studies on import as “Possible RCT” or “Not RCT,” with 99% accuracy in identifying non-RCTs. With Covidence as an integral part of our current service model, it is important to maintain awareness of these updates to existing technologies and new technologies entering the market. It's also important to remember the potential increase in costs that these tools can mean for an SR service. It remains to be seen what kind of additional budgetary effect SR automation tools will have on our SR service if they are outside of our currently subscribed software.

A key takeaway that our SR service team has learned over the past 11 years is the necessity of adaptation and change. Our service offerings and outputs look very different now than they did in 2011, as seen in our in-house documentation data and changes in the field. We do not have a formal evaluation/feedback form for users of our service; however, we have not yet seen this as a limitation of our service. Using data from the past 11 years has allowed us to better understand and address issues regarding completion, librarian time commitments and work allocation, and request patterns. While each user population is unique, we hope other SR services can use our experience to inform their own workflows.

## Data Availability

Data associated with this article, including Excel documentation spreadsheet, are available upon request.
